# Myofascial force transmission between the calf and the dorsal thigh is dependent on knee angle: an ultrasound study

**DOI:** 10.1038/s41598-023-30407-3

**Published:** 2023-03-06

**Authors:** Lisa Mohr, Lutz Vogt, Christian Thiel, Michael Behringer, Jan Wilke

**Affiliations:** 1 Department of Sports Medicine and Exercise Physiology, Institute of Sports Sciences, Goethe University Frankfurt, Frankfurt am Main, Germany; 2 Division of Physiotherapy, Department of Applied Health Sciences, Hochschule für Gesundheit (University of Applied Sciences), Bochum, Germany; 3 Division of Health and Performance, Institute of Occupational, Social and Environmental Medicine, Goethe University, Frankfurt am Main, Germany; 4 Department of Movement Sciences, University of Klagenfurt, Klagenfurt am Wörthersee, Austria

**Keywords:** Physiology, Musculoskeletal system

## Abstract

A recent in-vivo experiment has shown that force can be transmitted between the gastrocnemius and the hamstring muscles due to a direct tissue continuity. However, it remains unclear if this mechanical interaction is affected by the stiffness of the structural connection. This study therefore aimed to investigate the impact of the knee angle on myofascial force transmission across the dorsal knee. A randomized, cross-over study was performed, including n = 56 healthy participants (25.36 ± 3.9 years, 25 females). On two separate days, they adopted a prone position on an isokinetic dynamometer (knee extended or 60° flexed). In each condition, the device moved the ankle three times from maximal plantarflexion to maximal dorsal extension. Muscle inactivity was ensured using EMG. High-resolution ultrasound videos of the semimembranosus (SM) and the gastrocnemius medialis (GM) soft tissue were recorded. Maximal horizontal tissue displacement, obtained using cross-correlation, was examined as a surrogate of force transmission. SM tissue displacement was higher at extended (4.83 ± 2.04 mm) than at flexed knees (3.81 ± 2.36 mm). Linear regression demonstrated significant associations between (1) SM and GM soft tissue displacement (extended: R^2^ = 0.18, p = 0.001; flexed: R^2^ = 0.17, p = 0.002) as well as (2) SM soft tissue displacement and ankle range of motion (extended: R^2^ = 0.103, p = 0.017; flexed: R^2^ = 0.095, p = 0.022). Our results further strengthen the evidence that local stretching induces a force transmission to neighboring muscles. Resulting remote exercise effects such as increased range of motion, seem to depend on the stiffness of the continuity.

Trial registration: DRKS (Deutsches Register Klinischer Studien), registration number DRKS00024420, first registered 08/02/2021, https://drks.de/search/de/trial/DRKS00024420.

## Introduction

The deep fascia not only provides sensory input^1–5^, but has been demonstrated to link both, parallelly (e.g., gastrocnemius and soleus muscles^6–8^) and serially (e.g., gastrocnemius and hamstring muscles^9^) arranged skeletal muscles. When considering serial connections, the resulting body-wide planes of structural tissue continuity have been referred to as myofascial chains^10^. Particularly, the dorsal myofascial chain (plantar fascia, Achilles tendon, gastrocnemius muscle, Hamstrings, sacrotubular ligament, lumbar fascia/M. erector spinae) has been thoroughly evaluated with regard to its morphology and in-vitro mechanics^9^. Of note, cadaver studies revealed that local tissue straining produces substantial amounts of force transmission between neighbouring components^11,12^. Altered in-series myofascial force transmission has, therefore, been suggested as a potential cause of orthopedic pain conditions^13^ and flexibility deficits^14–17^. Yet, the ecological validity of findings from cadaver studies for in vivo conditions is limited as no active muscle activity as well as no gravity effects can be observed during related experiments.

To date, only a few studies have investigated the mechanical relevance of myofascial chains in the living organism. Most of the available research did not directly quantify the amount of transmitted force within myofascial chains but measured functional parameters such as range of motion (ROM). For example, some studies found non-local improvements in ROM after stretching or self-massage of structurally connected, distant body locations^12,15–18^. However, these functional results can only be considered as indirect evidence but not as a valid proof of force transmission. Against this background, a pilot study of Wilke et al.^19^ investigated the effects of a passively induced ankle movement (dorsal extension) at extended knees on tissue displacement in the dorsal thigh. The rationale behind this approach was that a local force transmitted through a myofascial connection should cause a visible displacement in the connected tissue. Indeed, simultaneous ultrasound visualization of the soft tissue revealed a caudal displacement of the semimembranosus (SM) muscle.

Despite the early evidence pointing towards force transmission in the living organism, the study by Wilke et al.^19^ had a small sample size and, more importantly, no control condition with flexed knee. Knowing the conditions (e.g. joint positions) under which force transmission occurs, however, is crucial when aiming to use or prevent it in sports, movement, and therapy. The present study addressed this deficit based on the following assumption: for force to be transmitted from the calf to the dorsal thigh, the tissue connection between the gastrocnemius muscle and the hamstring muscles needs to be stiffened by extending the knee. Anatomically, the gastrocnemius muscle attaches to the medial and lateral epicondyles of the femur^20^. Due to the resulting biarticularity, extension of the knee results in a higher stretch of the gastrocnemius muscle compared to the flexed knee^21^. Because the gastrocnemius muscle and its fascial connection to the hamstring muscles are stretched and tightened during knee extension, whereas they lose tension during knee flexion, higher force transmission can be expected with the knee extended.

Consequently, our study aimed to investigate the hypotheses that (1) passive dorsal extension of the ankle causes a caudal displacement of the hamstring muscles when the knee is extended, whereas little or no displacement is observed when the knee is flexed, and that (2) the extent of the Hamstrings displacement depends on the extent of ankle movement.

## Methods

### Design & ethics

The study adopted a randomized crossover design. All participants underwent two experimental conditions, which were separated by a washout period of 24 h. Displacement of the semimembranosus and its deep fascia upon ankle joint motion was examined with the knee in (1) extended and (2) flexed position. All assessments were performed at the same daytime, in the same room, and by the same investigators. The trial was conducted according to the Guidelines of Good Clinical Practice^22^ and according to the Declaration of Helsinki including its recent modification of Fortaleza. The local ethics committee of Goethe- University Frankfurt (2020-71G) provided ethical approval and all participants provided written informed consent. Written informed consent for publication was obtained by all individual persons shown in any images or videos. The trial was first registered on 08/02/2021 in DRKS (Deutsches Register Klinischer Studien, registration number DRKS00024420, https://drks.de/search/de/trial/DRKS00024420).

### Sample

A total of 56 healthy individuals (25♀, 31♂; 25.36 ± 3.9 years; BMI 23.0 ± 2.8) aged between 18 and 40 years were recruited. According to Faul et al.^23^, an a priori biometric sample size calculation was performed, using the algorithm of G*Power, version 3.1.5 (Heinrich-Heine University of Düsseldorf, Germany) for each of the two hypotheses. The computation showed hypothesis one (dorsal extension of the ankle at extended knees causes a higher displacement of SM than ankle movement at flexed knees) to require n = 29 individuals (α = 0.05, 1 − β  =  0.8, Cohen’s d = 0.5, Dropout: 5%). Hypothesis two (SM displacement depends on ankle movement/GM displacement) yielded a minimum of n = 56 participants (α = 0.05, 1 − β  =  0.8, Cohen’s f^2^ = 0.15, Dropout: 5%). Consequently, the enrollment of n = 56 individuals ensured sufficient power to detect both, significant differences (hypothesis 1) and associations (hypothesis 2) with medium effect sizes according to Cohen^24^.

Flyer and poster advertising as well as personal addressing were used for recruitment. Exclusion criteria were defined as orthopedic, cardiovascular, neurological, endocrine and psychiatric diseases, acute inflammation, intake of drugs that modify pain perception and proprioception, presence of delayed onset muscle soreness, pregnancy or nursing period, and history of surgery in the lower limb.

## Experimental approach

Participants layed in a prone position on an isokinetic dynamometer (IsoMed 2000, D. & R. Fersti GmbH, Hemau, Germany). Based on the neutral position of the ankle joint, the ankle from the randomly chosen leg was set in 25° plantarflexion to obtain a standardized starting point of passive motion. It was then moved passively from the starting position to the maximal achievable dorsal extension using the continuous passive function of the dynamometer. After a warm-up of three plantar flexion–extension cycles^11^, three repetitions with a constant angular velocity of 5°/s^19,25–27^ were conducted.

**Figure 1:**
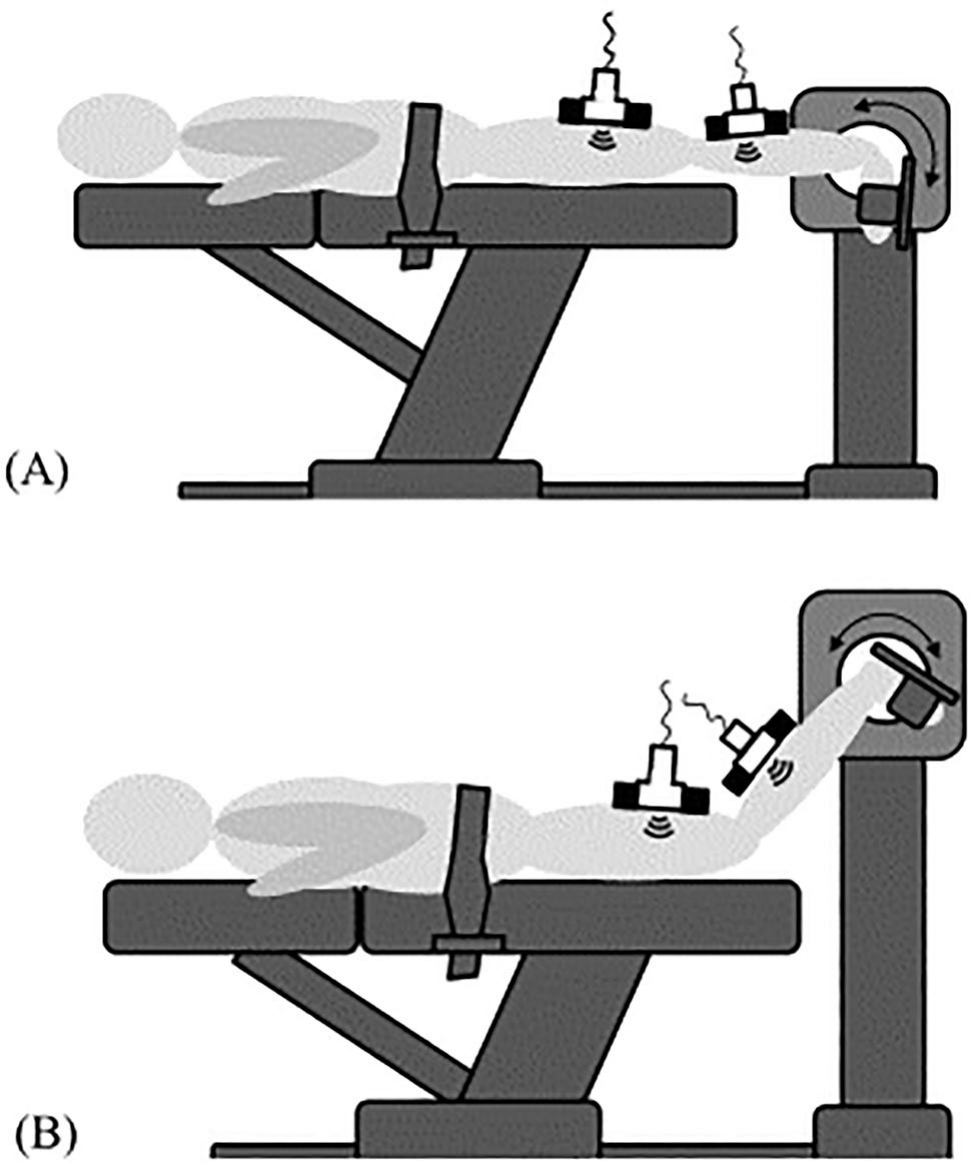
Schematic visualization of the experimental approach. Participants underwent two conditions: in randomized order, in prone position, the ankle was moved between plantar flexion and dorsal extension by use of a dynamometer (arrows) with the knee in extended (**A**) and flexed (**B**) position. Ultrasound recordings of the calf and dorsal thigh were made to estimate tissue displacement induced by ankle motion.

All participants underwent the assessment procedure twice with a wash out period of 24 h: in one session, the knee was fixed in extension (0° knee angle) while in the other condition, the knee was fixed in 60° flexion. The order of the two conditions was determined using balanced randomization. The 60° angle in the flexed position was chosen, because the gastrocnemius has been shown to be slack at this point and hence, no or only small magnitudes of force transmission can be expected^28^. To minimize secondary movement, a velcro strap was fixed at the participants pelvis (Fig. 1) while special care was given not to compress the dorsal thigh’s soft tissue. During the experiment, the participants were instructed to avoid voluntary muscle activity, which was verified using surface electromyography (sEMG, BioNomadix EMG2, Biopac Systems Inc., Goleta, CA, United States). Four electrodes (EL503 general- purpose electrodes, Biopac Systems Inc., Goleta, CA, United States) were placed on the SM and the gastrocnemius medialis (GM) muscles (two each) according to the SENIAM (surface EMG for non-invasive assessment of muscles) recommendations. If the position of the electrodes would overlap with the position of the transducer, the electrodes were slightly displaced.

**Figure 2:**
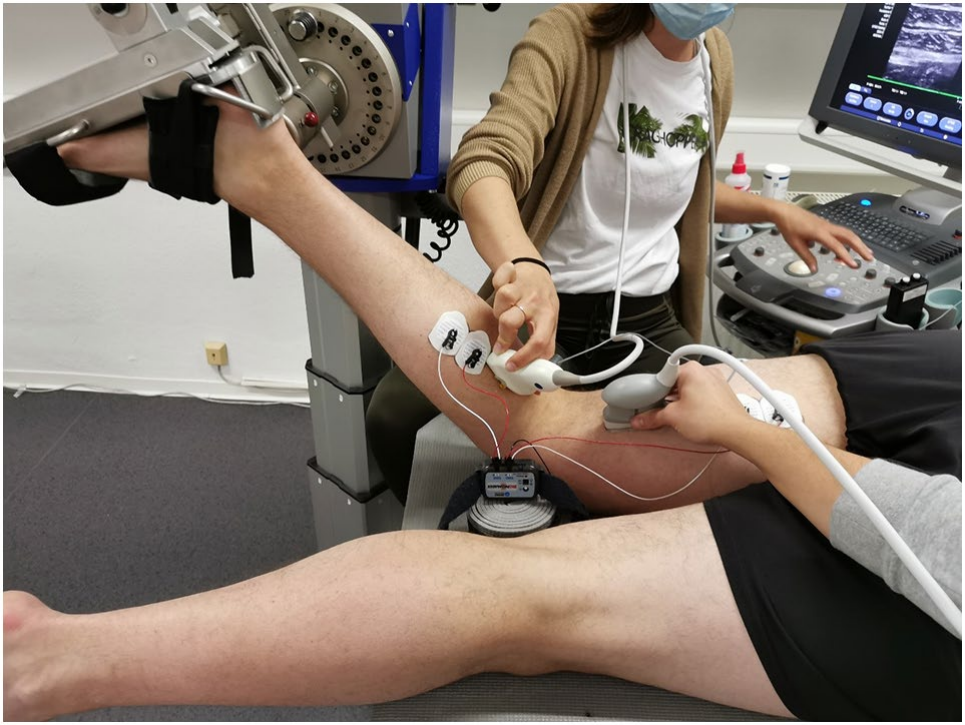
Exemplary measurement with the knee 60° flexed. In prone position, the ankle was moved three times between plantar flexion and dorsal extension, using the continuous passive function of the dynamometer. Ultrasound recordings of the gastrocnemius medialis and the semimembranosus were made to estimate tissue displacement induced by ankle motion. Electromyography was used to confirm muscle inactivity.

Tissue displacement during passively induced ankle motion was visualized using video recordings with two high-resolution ultrasound devices (Siemens Acuson X300 PREMIUM EDITION, Siemens Medical Solutions USA, Inc., Mountain View, CA and Siemens Acuson Redwood, Siemens Healthcare GmbH, Erlangen, Germany). Linear array transducers (VF10-5 linear array, 5.0–10.0 MHz, Siemens Medical Solutions USA, Inc., Mountain View, CA and 10L4 linear array, 3.3–11.4 MHz, Siemens Healthcare GmbH, Erlangen, Germany) were placed over (1) the GM and (2) the SM muscle. In detail, the transducers were positioned on the proximal third of the muscle belly of the GM and on the distal third of the SM. US transducer locations as well as electrode locations were marked on the skin with a permanent marker to ensure identical measurement sites between the two conditions. Participants were instructed to renew the markings if necessary. A schematic illustration of the experimental approach is shown in Fig. 1; Fig. 2 displays an exemplary measurement with the knee at 60° flexed position.

### Outcomes

The maximal horizontal displacement (mm) of SM and GM was quantified using cross-correlation analysis of the recorded US videos. The used MATLAB—(The MathWorks, Inc., Natick, MA, United States) based algorithm uses pre-defined determined regions of interest (ROI) to determine the correlation coefficients between pixel grey levels of successive frames^29^. The algorithm has been shown to represent a reliable method to quantify tissue displacement (ICC 0.7–0.99)^29^.

**Figure 3:**
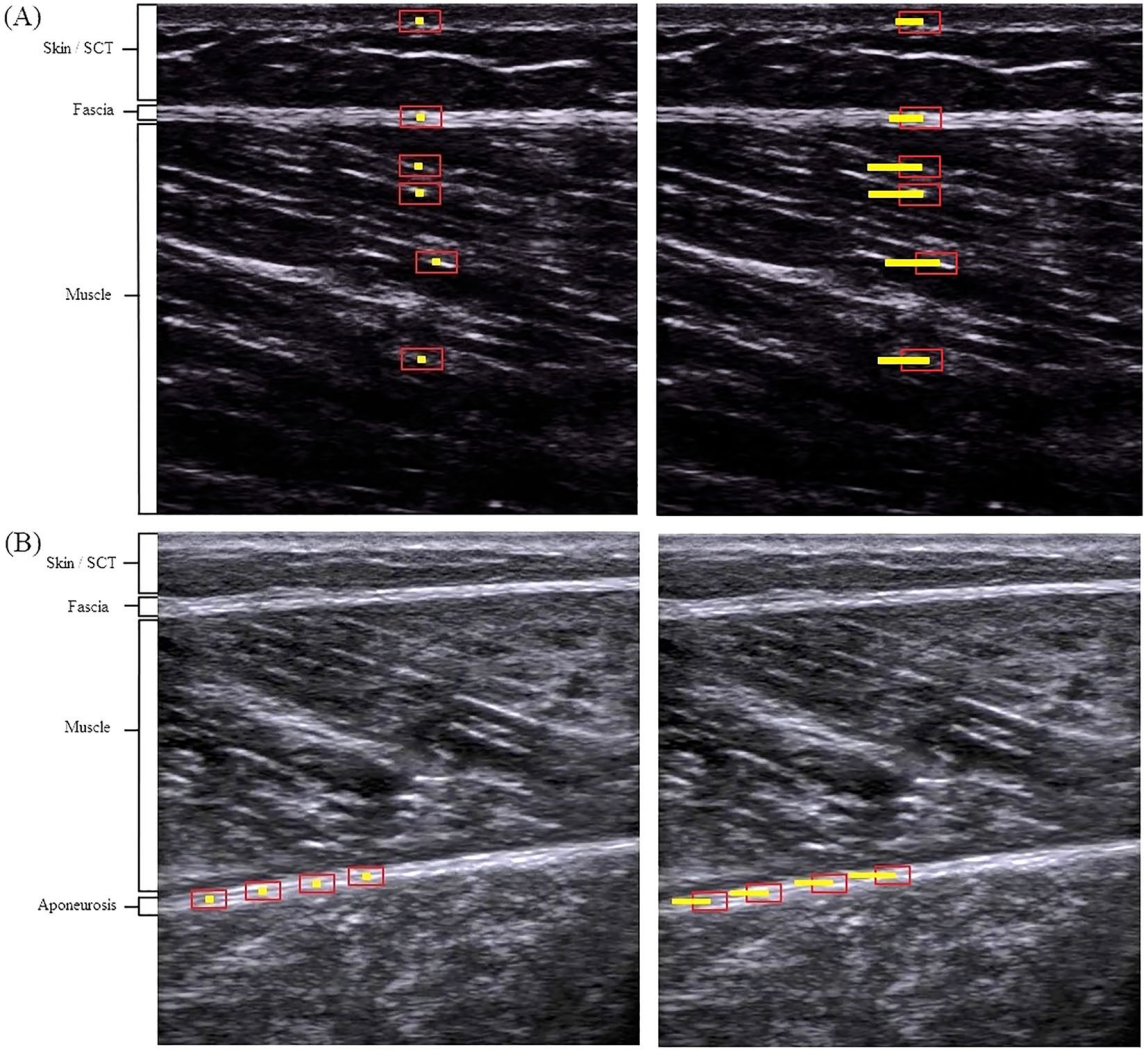
Exemplary visualization of the ultrasound cross-correlation analysis in (**A**) semimembranosus and (**B**) gastrocnemius medialis muscle. (**A**) In the semimembranosus muscle, six ROIs (regions of interest; red rectangles) have been positioned at rest (left image): one in each the subcutaneous tissue (SCT) and the fascia, and four within the muscle. Easily visible and traceable fibres in the centre of the image were selected as equidistant as possible. (**B**) In the gastrocnemius medialis muscle, four ROIs (red rectangles) were positioned in the inferior aponeurosis from the left toward the centre of the image. For both, (**A,B**) the small yellow squares within the ROIs represent the centres of the ROIs before movement occurred. Upon movement (right images), pixel displacements relative to the centre of the non-moving ROIs are tracked (yellow line in right pictures). The end of the line indicates the maximal displacement as computed by the software algorithm.

To analyze the tissue displacement of the SM, six equidistant ROIs (approximate size: 5 × 1 mm) were selected in the middle of the US image^11,19^: four in the muscle tissue and one ROI in each, the subcutaneous tissue and the deep fascia (Fig. 3). To analyze tissue displacement of the GM, four equidistant ROI's were selected in the inferior aponeurosis, from the left towards the center of the US image (Fig. 3). Excellent reliability of this approach has been demonstrated^19,27^. A video of an exemplary analysis can be found in the supplementary material.

### Data processing and statistics

US data and ROM measurements of the dynamometer, as well as EMG data, were synchronized using a common digital interface (AcqKnowledge, Biopac Systems Inc., Goleta, CA, United States). Unwanted muscle activity was detected using the internal function of the used software package (AcqKnowledge, Biopac Systems Inc., Goleta, CA, United States). Specifically, under resting conditions, the mean and standard deviation of the EMG signal were determined over a 0.25 s period. Based on the algorithm of Hodges and Bui^30^, a filtered average rectified value (ARV) was computed. The variance with regard to the noise was then extracted dividing the difference between the ARV and the mean by the standard deviation. For the resulting signal, the median was calculated for the entire waveform and any activity exceeding this median for at least 0.1 s was considered as muscle activity and the corresponding repetition was excluded from analysis. With confirmed muscle inactivity, the mean values of the three repetitions were calculated for the dynamometer (ankle angle in °) and ultrasound data (horizontal displacement of the tissue in mm) for both conditions (knee extended/60° flexed), respectively.

A paired t-test was performed in order to reveal systematic differences between the two testing conditions, thus evaluating the first hypothesis (higher fascial displacement in the condition with the knee extended). Effect sizes of significant differences were calculated and interpreted according to Cohen^24^ as small (d ≥ 0.2), medium (d ≥ 0.5) or large (d ≥ 0.8).

For the second hypothesis (possible relationship between ankle motion and displacement of dorsal thigh’s soft tissue), we used linear regression for parametric data and linear regression with bootstrapping for non-parametric data with BCa (Bias Corrected and accelerated) procedures. The dependent variable was displacement of the soft tissue of the SM and ankle ROM / GM aponeurosis displacement were selected as the predictors. Again, effect sizes were calculated according to Cohen^24^ and interpreted as weak (f = 0.1), medium (f = 0.25), and strong (f = 0.4). All calculations are performed using SPSS 20 (IBM, Armonk, USA); the significance level was set at α = 0.05.

## Results

All 56 participants completed the study, but data of one participant had to be excluded from analysis due to involuntary muscle activity. In the 55 analyzed participants (24♀, 31♂; 25.38 ± 3.93 years; BMI 23.1 ± 2.8; 6.2 ± 3.9 h exercise/week), the mean ankle ROM was 26.02 ± 6.15° with the knee extended and 28.22 ± 7.41° with the knee flexed. Displacement of the SM was 4.83 ± 2.04 mm while the knee was extended and 3.81 ± 2.36 mm while the knee was flexed. The mean GM displacement was 24.36 ± 7.58 mm with the knee extended and 21.43 ± 6.87 mm with the knee flexed. The paired t-test showed that SM displacement was significantly higher with the knee extended than with the knee flexed (t(54) = 3.285, p = 0.002, d = 0.44). Similarly, GM displacement was higher with the extended knee than with the flexed knee (t(54) = 4.091, p < 0.001, d = 0.55).

Linear regression (Table 1) revealed significant associations between GM and SM (dependent variable) displacement for both conditions (knee extended: R^2^ = 0.182, p = 0.001; knee flexed: R^2^ = 0.168, p = 0.002). Similarly, although with a smaller effect size, ankle ROM predicted SM displacement (knee extended: R^2^ = 0.103, p = 0.017; knee flexed: R^2^ = 0.095, p = 0.022).

**Table 1:** Results of linear regression analysis.

Condition	Variable	b (95% CI)	SE	Beta	T	p	f
knee extended	SM displacement	2.022 (0.31, 3.74)	0.85		2.37	0.02	0.47
	GM displacement	0.115 (0.05, 0.18)	0.03	0.427	3.44	0.001	
	SM displacement^a^	−0.435 (−3.82, 3.0)	1.75		−0.20	0.84	0.34
	ROM^a^	0.103 (0.04, 0.17)	0.04	0.321	2.47	0.02	
knee flexed	SM displacement^a^	0.808 (−1.06, 2.69)	0.89		0.84	0.41	0.45
	GM displacement^a^	0.141 (0.05, 0.23)	0.05	0.410	3.27	0.002	
	SM displacement^a^	−1.443 (−5.51, 3.01)	2.03		−0.64	0.53	0.32
	ROM^a^	0.1 (0.03, 0.17)	0.04	0.308	2.36	0.02	

Semimembranosus (SM) displacement (dependent variable) was predicted by gastrocnemius medialis (GM) displacement and ankle range of motion (ROM) with extended/flexed knee.

*b* regression coefficient, *CI* confidence interval, *SE* standard error, *f* Cohen’s effect size^24^.

^a^Bootstrapped analysis: confidence intervals and standard errors obtained with BCa-bootstrapping (10,000 BCa samples).

## Discussion

The mechanical relevance of the extramuscular connective tissue (i.e. the deep fascia) represents one of the main topics of fascia research^31^. However, so far, the evidence on in-series myofascial force transmission had mainly been derived from cadaver experiments and studies with functional outcomes^11,14–16^ while valid proxies of force transmission were rarely used. Cruz-Montecinos et al.^25^ and Wilke et al.^19^ provided first evidence pointing towards mechanical interactions between serially connected muscles in the living organism. Cruz-Montecinos et al.^25^ found a ventral pelvic tilt to lead to a cranial displacement of the gastrocnemius fascia. However, the observed force transmission from cranial to caudal contrasts with the caudal-cranial direction of weight-bearing movements in everyday life (e.g., walking, running, jumping). In their pilot study, Wilke et al.^19^ investigated mechanical interactions in the caudal-cranial direction, observing a force transmission from the gastrocnemius muscle to the SM during passive stretching of the calf. Yet, neither Wilke et al.^19^ nor Cruz-Montecinos et al.^25^ used a control condition.

Against this background, the present study is the first demonstrating caudal-cranial in-vivo interactions of serially connected muscles in a randomized, controlled design. Our results show that the knee angle can have a relevant impact on soft tissue displacement following non-local movement. As expected, there was significantly less soft tissue displacement of the SM with the knee flexed (3.81 ± 2.36 mm) when compared to the extended knee (4.83 ± 2.04 mm). The results observed in the extended knee condition are basically consistent with the findings of Wilke et al.^19^ and earlier trials reporting non-local improvements in ROM following exercise in distant joints^15–18^.

Our findings have significant implications for clinical practice. As indicated, the here presented data strengthen the evidence that local stretching has a mechanical impact on neighboring body parts. While this had already been demonstrated^15–18^, we showed that the magnitude of remote exercise effects stemming from force transmission (i.e., ROM increases in adjacent joints), may substantially depend on the stiffness of the affected tissue continuity. The gastrocnemius muscle is in a slack position when the knee is flexed^21,28^ and hence, the strain produced by ankle movement is first used to bring the muscle to tension before it can transmit force to the knee. In contrast, if the knee is extended, the tissue continuity at the dorsal knee is pre-tensioned and able to directly transmit force from the calf to the thigh. Coaches, therapists, and practitioners designing and selecting stretching treatments can benefit from the new knowledge about the varying degrees of force transmission because it strongly affects the choice of exercise positions. For instance, if the goal of ankle stretching would be to influence the dorsal thigh, the fascial connection should be brought into a pre-stretched position (extended knee) to maximize effectiveness. In general, these thoughts could imply a paradigm shift, moving from targeting individual muscles towards involving complete myofascial chains. With regard to the example above, Hamstring flexibility could not only be improved by stretching these muscles or the gastrocnemius but also by involving the lower back region, which is connected to the dorsal thigh via the sacrotuberous ligament^12^. In addition to increasing stretch intensity (local plus non-local strain vs. local strain only), a holistic, chain-based approach may safe time owing to the simultaneous work on multiple structures. Another application refers to the prevention and treatment of orthopaedic complaints. Achilles tendinopathy is associated with a thickened plantar fascia^32^, plantar fasciitis^33,34^ has been shown to be associated with increased Hamstrings stiffness and sacroiliac pain correlates with altered activation of the latissimus dorsi muscle^35,36^. Possibly, these non-local symptoms represent a result of altered force transmission and may be resolved or alleviated by focussed treatments based on myofascial chains.

Besides investigating the influence of the knee position on force transmission, our study also showed that the displacement of the GM aponeurosis is a better predictor of SM displacement than ankle ROM. This is plausible because ROM does not only depend on structural (e.g., stiffness) but also on neural (e.g., stretch tolerance) factors^37,38^. Nevertheless, the question arises as to why only a relatively small percentage of the variance could be explained by both ankle ROM and GM displacement. With regard to ROM, as indicated, this may be due to the involvement of neural factors. A possible explanation for the relatively small variance in GM displacement could be the different stiffness of the structures involved. Liu et al.^39^ found that dorsal extension of the ankle leads to inhomogeneous changes in the stiffness of muscles, tendons and fasciae of the calf. As stiffness is crucial for force transmission, the degree of force transmission may be subject to substantial variation and may not be linear with local tissue displacement. Another possible hypothesis relates to the fact that the gastrocnemius does not only have proximal connections to the semimembranosus muscle. Anatomical research, in addition, identified a fascial connection to the semitendinosus muscle^9^ and therefore, force transmission will most likely be distributed between the SM and the semitendinosus muscle. In sum, future studies should be geared to investigate the relevance of stiffness variations as well as the relative contributions of different muscles in taking up forces transmitted through myofascial connections.

Some methodological considerations should be made when interpreting the results of our study. First, measurements of soft tissue displacement represent a highly plausible surrogate of force transmission, but not a direct measurement of force. Furthermore, we defined maximum ankle dorsal extension as the subjective maximum tolerated by the participants, which caused ROM to vary in the two starting positions (26.02° ± 6.15° with the knee extended; 28.22° ± 7.41° with the knee flexed). Also, with the knee flexed, the participants tended not to report a maximum stretch sensation of the calf muscles but rather a blocking sensation in the ankle joint. Again, this can be explained by the biarticularity of the gastrocnemius muscle: when the knee is flexed, the muscle does not reach a fully stretched position^21^.

## Conclusion

The present study is the first to show the influence of the knee angle on in-series myofascial force transmission between the ankle and thigh under in-vivo conditions. Besides improving the understanding of the function of the myofascial system, this finding may serve as a basis for the selection and design of exercises when aiming to maximize effectiveness or treat locomotor complaints.

## Supplementary Information

Supplementary Legends.

Supplementary Video 1.

## Data Availability

The datasets used and/or analyzed during the current study are available from the corresponding author on reasonable request.
