# Supplementary material for: Myofascial force transmission between the calf and the dorsal thigh is dependent on knee angle: an ultrasound study
Source: Sci Rep. 2023 Mar 6;13:3738. doi: 10.1038/s41598-023-30407-3 (PMC9988973; doi:10.1038/s41598-023-30407-3)
Supplement: Supplementary file 1 — Supplementary Legends. [file 41598_2023_30407_MOESM1_ESM.docx]

Supplementary video legends

**Video 1.** Exemplarily analysis of tissue displacement. Using Motion 2018 (Six ROIs (regions of interest, blue rectangles) were manually selected in the middle of the US (ultrasound) image: one ROI in each, the subcutaneous tissue and the deep fascia and four in the muscle tissue. The yellow squares within the ROIs represent the centres of the ROIs before movement occurred. Upon movement, pixel displacements relative to the centre of the non-moving ROIs are tracked (white squares). Using the differences, the software algorithm computes the maximal displacement throughout the frames. The line at the bottom of the US image indicates the beginning of the ankle movement.
